# Performance of Cross‐Validated Targeted Maximum Likelihood Estimation

**DOI:** 10.1002/sim.70185

**Published:** 2025-07-17

**Authors:** Matthew J. Smith, Rachael V. Phillips, Camille Maringe, Miguel Angel Luque‐Fernandez

**Affiliations:** ^1^ Department of Medical Statistics London School of Hygiene and Tropical Medicine London UK; ^2^ Department of Biostatistics, School of Public Health University of California at Berkeley Berkeley California USA; ^3^ Inequalities in Cancer Outcomes Network London School of Hygiene and Tropical Medicine London UK; ^4^ Department of Statistics and Operations Research University of Granada Granada Spain

**Keywords:** causal inference, data sparsity, Donsker class condition, epidemiology, near‐positivity violation, observational studies, targeted maximum likelihood estimation

## Abstract

**Background:**

Advanced methods for causal inference, such as targeted maximum likelihood estimation (TMLE), require specific convergence rates and the Donsker class condition for valid statistical estimation and inference. In situations where there is no differentiability due to data sparsity or near‐positivity violations, the Donsker class condition is violated. In such instances, the bias of the targeted estimand is inflated, and its variance is anti‐conservative, leading to poor coverage. Cross‐validation of the TMLE algorithm (CVTMLE) is a straightforward, yet effective way to ensure efficiency, especially in settings where the Donsker class condition is violated, such as random or near‐positivity violations. We aim to investigate the performance of CVTMLE compared to TMLE in various settings.

**Methods:**

We utilized the data‐generating mechanism described in Leger et al. (2022) to run a Monte Carlo experiment under different Donsker class violations. Then, we evaluated the respective statistical performances of TMLE and CVTMLE with different super learner libraries, with and without regression tree methods.

**Results:**

We found that CVTMLE vastly improves confidence interval coverage without adversely affecting bias, particularly in settings with small sample sizes and near‐positivity violations. Furthermore, incorporating regression trees using standard TMLE with ensemble super learner‐based initial estimates increases bias and reduces variance, leading to invalid statistical inference.

**Conclusions:**

We show through simulations that CVTMLE is much less sensitive to the choice of the super learner library and thereby provides better estimation and inference in cases where the super learner library uses more flexible candidates and is prone to overfitting.

## 1 Introduction

In public health research, it is often of interest to assess the causal relationship between an exposure or treatment and an outcome. Examples include the causal effect of immunotherapy on the probability of survival after cancer diagnosis, the effect of smoking on rheumatoid arthritis, or the effect of childhood adversities on mental health later in life. Estimates of these relationships are often learned from real‐world data and can be complex to ascertain, requiring machine‐learning estimators, or be biased, such as spurious associations if there are factors that influence both the treatment and outcome variables. Randomized controlled trials (RCTs) remove confounding due to randomization of individuals to treatment groups. However, RCTs are not always feasible, such as for ethical reasons, or the randomization process may fail. When causality cannot be guaranteed by design, such as in observational studies, causal inference methods based on the g‐formula should be used when the research question claims causality, or to improve adjustment for confounding [1].

Methods used to estimate these causal effects can be broadly categorized into those that estimate the exposure model based on propensity scores [2, 3, 4, 5], outcome model based on g‐computation [6, 7, 8], or doubly robust methods that estimate both exposure and outcome models [9, 10, 11]. There are some exceptions, such as proximal causal inference using negative control variables for non‐parametric identification of causal effects in the presence of hidden confounding bias or settings where exchangeability does not hold [12]. Doubly robust methods are so named because they are consistent estimators of the causal effect as long as at least one of the two models is correctly specified. For causal effect estimation with machine learning, doubly robust methods bring faster convergence rates, assuming both nuisance models are correctly specified [13, 14]. Of the doubly robust methods, targeted maximum likelihood estimation (TMLE) has been shown to consistently provide the least biased estimate of the causal effect in comparison to other doubly robust methods such as inverse probability treatment weighting with regression adjustment (IPTW‐RA) or augmented inverse probability treatment weighting (AIPTW) [11]. The advantages of TMLE have been demonstrated theoretically, and in numerous simulation studies and applied analyses [15, 16]. However, it is worth noting that TMLE is not the only valid approach for doubly robust methods; others exist, such as the Double‐Debiased Machine Learning algorithm [17]. As a plug‐in estimator, TMLE respects the global limits of the statistical model (e.g., limiting the possible range of the targeted parameter). TMLE reduces bias through the use of ensemble and machine‐learning algorithms, and it has the minimum asymptotic variance in the class of semiparametric estimators. Statistical inference may be based on the efficient influence curve (IC) or a targeted bootstrap [11, 18, 19, 20, 21]. The TMLE algorithm is generally applicable for a wide range of causal estimands, such as time‐varying effects, dynamic treatment regimes, and mediation analysis, among others. However, we focus only on point‐treatment effects and the use of TMLE in estimating the average treatment effect (ATE).

Oftentimes, the TMLE framework considers data‐adaptive ensemble machine learning algorithms for estimation of nuisance models (i.e., the outcome and treatment models) [22]. The Influence Curve (IC) and the functional Delta Method, seen as an extension of the Central Limit Theorem for functionals, are used in the targeting step of the TMLE and to compute Wald‐type confidence intervals. This assumes that the remainder term, generated when examining the difference between the TMLE estimator and the truth, is a sample average of a quantity converging to 0 in probability. The Donsker condition exists for both the nuisance functions and their estimators, so TMLE's consistency and asymptotic normality rely on both of these being Donsker. This is valid and provides a valid inference if the nuisance models satisfy the Donsker class condition, that is, that they are not highly flexible machine learning algorithms that are prone to overfitting.

In our setting, given the bias correction step, the data is used twice: To estimate (i) the nuisance functions and (ii) the bias reduction. Donsker class implies that the estimator of the nuisance functions are not too complex, including smooth parametric models, but also bounded monotone functions and smooth functions with bounded partial derivatives, to avoid overfitting [23]. This can become overly restrictive when we use arbitrarily flexible machine learning algorithms to estimate the nuisance parameters (i.e., Lasso, Random Forest, Boosting, etc.), which are prone to overfitting and break the Donsker class condition. In situations where the Donsker class condition is violated, the variance is anti‐conservative, leading to confidence intervals with poor coverage [14, 24]. Cross‐validation or cross‐fitting of the TMLE algorithm (CVTMLE) is a simpler, yet effective, way to ensure efficiency, especially in settings where the Donsker class condition is violated [14, 25]. Cross‐validation is a statistical learning technique widely used in regression and classification problems to avoid over‐fitting and improve the asymptotic consistency and efficiency of estimations [26].

There are a couple of approaches to CVTMLE. One approach is based on Zheng & van der Laan [27] who propose cross‐validating the entire TMLE algorithm and averaging all estimated treatment effects and their variances, denoted here as CVTMLE[all]. More recently, Levy (2018) suggested that cross‐validating the initial outcome and exposure models (which we denote as CVTMLE[Qg]) would be sufficient for a more computationally efficient estimation of the target parameter, while retaining the theoretical properties of TMLE, particularly in cases where more complex machine learning algorithms are required [28]. We also propose to relax the Donsker condition on the outcome process only, CVTMLE[Q], which corresponds to settings where the process leading to assignment of the exposure or treatment is simple or known, such as in clinical trials.

We aim to investigate the performance of CVTMLE[all], CVTMLE[Qg], and CVTMLE[Q] compared to TMLE in settings with varying degrees of violation of the Donsker class condition. In Section 2, we describe TMLE and its cross‐validated versions. In Section 3, we outline the simulations of different settings likely violating the Donsker class condition. In Section 4, we report the respective performances of TMLE and CVTMLE when using different SuperLearner libraries. In Section 5, we propose an empirical example from the medical literature, and in Section 6, we reflect on the meaning of our results for practice and provide specific guidance.

## 2 Methods

### 2.1 Targeted Maximum Likelihood Estimation

TMLE is a plug‐in, semi‐parametric, doubly robust method that reduces the bias of an initial estimate by allowing for flexible estimation using parametric or nonparametric data‐adaptive machine‐learning methods to target an estimate closer to the true model specification [11]. Several tutorials for TMLE have been published along with a systematic review describing its applications [1, 16, 29, 30, 31].

TMLE is described in the *Targeted Learning* book by van der Laan and Rose [18]. We briefly outline the algorithmic steps when using TMLE for the average treatment effect (ATE) here. Given the data structure O=(W,A,Y) observed on *n* individual records, where **W** represents a set or vector of confounders, A is a binary treatment or exposure mechanism, and Y is the outcome, we suppose our target parameter is the ATE, across individuals. Using the potential outcomes framework, each individual has two potential outcomes: The outcome that would have been observed had the individual been exposed (A=1) denoted as Y(1), and the outcome that would have been observed had the individual not been exposed (A=0) denoted as Y(0).

To deal with a continuous outcome Y, the TMLE framework transforms linearly the outcome within [0, 1] as follows: $Y^{\prime }=(Y-a)/(b-a)$, where b and a are respectively the maximum and minimum values observed for Y. Then, the ATE is estimated on the transformed outcome $Y^{\prime }$, as usual, but the original limiting normal distribution and confidence intervals are obtained after multiplying by (b−a) to get the ATE in the original scale.

#### 2.1.1 Step 1: Predict the Outcome

TMLE fits the outcome model (i.e., $Q^{0}(A,W)=E(Y|A,W)$) using the observed values of the outcome, given observed treatment A and covariates W. To minimize model misspecification, an ensemble of machine‐learning algorithms (i.e., Super Learner) can be used to estimate E(Y|A,W). Super Learner uses cross‐validation to find the best‐fitting combinations of parametric and non‐parametric models from a range of machine‐learning algorithms to provide initial predictions of the outcome for each individual i (i.e., $Q_{i}^{0}(A,W)$) [18, 19].

#### 2.1.2 Step 2: Predict the Treatment

A Super Learner, an ensemble of—potentially different—machine learning algorithms, can also be used to fit the propensity score model for the treatment (i.e., g(A,W)=P(A=1|W)) and to predict treatment for each individual i [18, 19].

#### 2.1.3 Step 3A: Calculate Clever Covariates

Clever covariates (i.e., H(A,W)) are calculated using information from the observed treatment and predictions from the propensity score model.

$H(A,W)=\frac{2A-1}{g(A,W)}$ for A=1 or A=0.

#### 2.1.4 Step 3B: Estimate the Fluctuation Parameter

The fluctuation parameter ($\epsilon =\{\epsilon_{0},\epsilon_{1}\}$) is estimated through a maximum likelihood procedure using weights. An intercept‐only model is fit using the observed outcome (Y) as the dependent variable with the logit of the initial prediction of $Q_{i}^{0}((A=1),W)$ as an offset and the regression model is weighted by the clever covariate, H(1,W). This process is repeated for A=0 so that two targeting models are fit [32, 33, 34].

When there is negligible remaining variability in $Y-Q_{i}^{0}(A,W)$, the fluctuation parameter will be estimated as close to 0.

#### 2.1.5 Step 4: Update the Initial Predictions of the Outcome

The fluctuation parameter is used to update the initial outcome predictions for each individual i from $Q_{i}^{0}(A,W)$ to $Q_{i}^{1}(A,W)$, optimizing the bias‐variance trade‐off for the targeted parameter (ATE):

For any $A=\{0,1\}:Q_{i}^{1}(A,W)=\text{expit}\left(\text{logit}\left(Q_{i}^{0}(A,W)\right)+\frac{\varepsilon_{A}}{g(A,W)}\right)$.

#### 2.1.6 Step 5: Estimate the Target Parameter

Plug in the updated estimates of the predicted outcomes to the target parameter mapping for the ATE:

$$\hat{\text{ATE}}=\frac{1}{n}\overset{n}{\underset{i=1}{\sum }}(Q_{i}^{1}\left(1,\;W\right)-Q_{i}^{1}\left(0,W\right))$$

#### 2.1.7 Step 6A: Estimate the Efficient Influence Curve

To calculate 95% confidence intervals for the ATE, TMLE requires an estimate of the standard error for the ATE. The standard error is estimated based on the efficient influence curve (IC), which characterizes variability and represents the most efficient function [18, 35, 36, 37]. The efficient IC identifies how much influence a single data point has on the performance of TMLE in estimating the ATE. It is given by:

$$\begin{array}{ll} {\text{IC}}_{\hat{ATE}} & =\left(\frac{A}{g(1,W)}-\frac{1-A}{g(0,W)}\right)\left(Y-Q^{1}(A,W)\right)\\ & \;+Q^{1}(1,W)-Q^{1}(0,W) \end{array}$$

The efficient IC combines information from the outcome model (Steps 1 and 4), the propensity score model (Step 2), and the estimate of the target parameter (Step 5) to account for the variability in the estimator.

#### 2.1.8 Step 6B: Estimate Standard Error

Then, the standard error (${\hat{\sigma }}_{\text{ATE}}$) for the ATE is evaluated as:

$${\hat{\sigma }}_{\text{ATE}}=\sqrt{\frac{\hat{Var}\left({IC}_{\hat{\text{ATE}}}\right)}{n}}$$

where $\hat{Var}\left({IC}_{\hat{\text{ATE}}}\right)$ is the sample variance of the IC of the estimated ATE.

#### 2.1.9 Step 6C: Calculate Confidence Intervals

The 95% confidence interval for the ATE is calculated as:

$$95\%\;\text{CI}\;=\;\hat{ATE}\;\pm \;z_{0.975}\left({\hat{\sigma }}_{\text{ATE}}\right)$$

### 2.2 Cross‐Validated Targeted Maximum Likelihood Estimation

TMLE is a doubly robust and efficient estimator, but is susceptible to performance issues when the initial estimator of the outcome or exposure model is too adaptive. In other words, if one or both of these initial estimators are overfit, then there is negligible residual variation remaining for the targeting step [25]. Combining cross‐validation with TMLE addresses this issue because training and validation are performed on independent sample subjects, which retains a realistic residual variation in the validation set.

There are several approaches to CVTMLE, each differing by what steps within TMLE are cross‐validated [27, 28]. All approaches start with K splits of the data. Each k (with k={1,…,K}) split defines each k fold, an indexing of the data into k sets for algorithm training and validation. For a K‐fold cross‐validation scheme, the data is split evenly into K subsets, the validation set for a given fold k ($V_{k}$) is defined by the data in subset k, and the data not in subset k is the training set for fold k ($T_{k}$). Each subject is part of one validation set and K−1 training sets.

**FIGURE 1:**
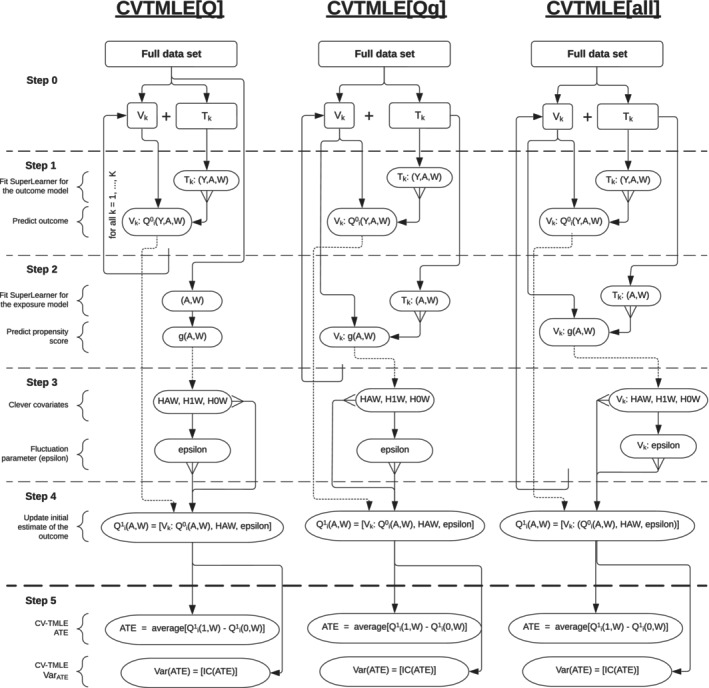
Process map of cross‐validated targeted maximum likelihood estimation.

We present three approaches to CVTMLE, (i) the original approach proposed by Zheng & van der Laan [27], (ii) one approach proposed by Levy [28], and (iii) an adaptation of Levy's approach. While Zheng & van der Laan propose to cross‐validate the entire TMLE process, denoted CVTMLE[all], Levy suggests that the calculation of the clever covariates and estimation of fluctuation parameter is done only once on the entire data, CVTMLE[Qg]. The third approach makes use of cross‐validation for estimating the outcome model only, and we denote this approach CVTMLE[Q]. The process for performing all three CVTMLEs is illustrated in Figure 1. All approaches impose that Step 1 of the TMLE algorithm described earlier is modified to accommodate K‐fold cross‐validation of the initial estimation of the outcome. For each cross‐validation scheme, k, (k={1,…,K}), estimate the outcome model (e.g., using the SuperLearner) using the training set, $Q_{i\in T_{k}}^{0}(A,\text{W})$. From this initial model, the outcome is predicted for all observations within the corresponding validation set, $Q_{i\in V_{k}}^{0}(A,\text{W})$. This process is repeated for each cross‐validation fold until each of the *n* observations in the original data set has a predicted initial outcome $Q_{i}^{0}(A,\text{W})$. In CVTMLE[Q], the rest of the algorithm, steps 2–6c, proceeds as in the standard TMLE algorithm. In CVTMLE[Qg], there is further cross‐validation of the initial estimation of the treatment process (step 2), and steps 3–6c proceed as in standard TMLE. Levy highlights that although predictions from the cross‐validated sets are stacked, CVTMLE[Qg] preserves the plug‐in characteristic of the TMLE estimator and performs well asymptotically [28]. CVTMLE[all] requires that steps 1–3 are cross‐validated.

## 3 Simulations

### 3.1 Setting

To evaluate the performance of TMLE and CVTMLE under near‐positivity violations, we perform a Monte Carlo simulation experiment in which we vary the likely severity of the violation of the Donsker class condition. There are different situations more likely to exacerbate violations of the Donsker class condition, such as: (i) data sparsity or small sample size, (ii) near‐positivity violations, and (iii) the use of highly data‐adaptive machine learning algorithms (e.g., tree‐based algorithms, such as random forests), all leading to non‐differentiability of the influence curve. Table 1 expands on these different scenarios leading to violation of the Donsker class condition and how the simulations were specified to replicate such scenarios.

**TABLE 1:** Settings where the Donsker class condition is likely violated and how these were reproduced in simulations.

Setting	Description	Impact on Donsker class condition	Simulation
Sample size	Small sample size requires a greater number of folds to be used within CVTMLE to allow a large enough training set.	Donsker class condition is based on asymptotic theory, which assumes that the sample size goes to infinity.	(i) Large sample size (n=1000) that does not require an increase in the number of folds (default of 10 folds is used).
		Small sample size can lead to random noise dominating the signal that machine learning algorithms are attempting to model.	(ii) Small sample size (n=200) that requires an increase in the number of folds, but is kept at the default of 10 folds.
Near‐positivity violation	There are groups of individuals with near‐zero probability to be treated or untreated, which leads to gaps in the data with unobserved or impossible combinations of the exposure/outcome.	Near‐positivity violations can introduce abrupt changes, discontinuities, or irregularities in the empirical process (i.e., estimation of the influence function), disrupting its smooth convergence.	(i) High prevalence of A (i.e., P[A=1]=0.8) created in exposure model.
			(ii) Extrapolation issue created by interaction in the outcome model between treatment and rare covariate.
Complex machine learning algorithms	Machine learning methods, such as tree‐based algorithms (e.g., random forests) used in the SuperLearner for the outcome and propensity score models	Tree‐based methods are highly data‐adaptive and have a tendency to overfit the data, especially in smaller sample sizes.	Using random forests with and without cross‐validation of TMLE to see the impact of cross‐validation on variance stabilization.
Non‐differentiability of the Influence curve (IC)	Influence curve must be continuous at every point in its domain, but fails to be differentiable at a bend, cusp, or vertical tangent.	IC is derived based on the limiting behavior of the estimator. When the Donsker class condition is violated, the empirical process does not converge to a smooth limiting distribution.	Combination of small sample size, near‐positivity violation, and complex machine learning algorithms used to estimate the target parameter.

### 3.2 Data‐Generating Mechanisms

We simulated scenarios of near‐positivity violations using data‐generating mechanisms described in Leger et al. [38].

First, we generated a vector of independent covariates $W=W_{1},W_{2},W_{3},W_{4},W_{5},W_{6},W_{7},W_{8}$, including six binary covariates following Bernoulli distributions with probabilities 0.1 for $W_{1}$, 0.4 for $W_{2}$, 0.7 for $W_{4}$, 0.5 for $W_{5}$, 0.3 for $W_{7}$, 0.8 for $W_{8}$, and two continuous covariates, $W_{3}$ and $W_{6}$ following a Gaussian distribution with mean 0 and standard deviation 1.

The exposure A was generated according to a Bernoulli distribution with probability obtained from a logistic regression model, using a logit link function, with the following linear predictor: $\alpha_{0}+\alpha_{1}W_{1}+\alpha_{2}W_{2}+\alpha_{4}W_{4}+\alpha_{6}W_{6}+\alpha_{7}W_{7}+\alpha_{8}W_{8}$. Where $\alpha_{0}$ was set to −0.45 or 1.05 to simulate the prevalence of exposed patients at 50% or 80%, respectively. $\alpha_{1}$, the coefficient for $W_{1}$ was set to log(5) to impose a near‐positivity violation particularly given that $W_{1}$ is generated with 10% prevalence. The rest of the coefficients, $\alpha_{2},\alpha_{4},\alpha_{6},\alpha_{7},\alpha_{8}$, were set to log(1.5).

Near‐positivity violation was determined from the values of the propensity scores (Appendix Table A1) that were greater than the cut‐off for truncation at 0.975. With 80% prevalence of the exposure there was, on average, 2.2 and 10.8 propensity scores that exceeded 0.975 for samples of 200 and 1 000, respectively. With 50% prevalence of the exposure, there were, on average, no propensity scores larger than the cut‐off for truncation.

**FIGURE A1:**
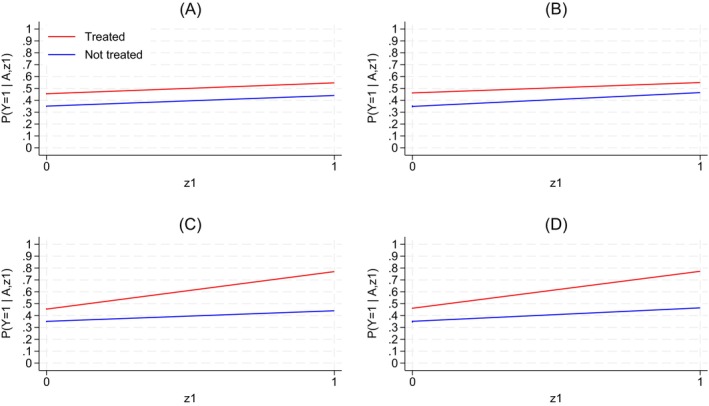
Probability of the outcome given the exposure and Z1 (variable creating near‐positivity violations), stratified by prevalence of the exposure (i.e., 50% or 80%) and presence of extrapolation issue (i.e., none or high). (A) is 50% prevalence of the exposure with no extrapolation issue. (B) is 80% prevalence of the exposure with no extrapolation issue. (C) is a 50% prevalence of the exposure with an extrapolation issue. (D) is an 80% prevalence of the exposure with an extrapolation issue.

The outcome was generated from a Bernoulli distribution with probability obtained from a logistic regression model, using a logit link function, with the following linear predictor: $-0.8+\beta_{A}A+\beta_{1}W_{1}+\beta_{2}W_{2}+\beta_{3}W_{3}+\beta_{4}W_{4}+\beta_{5}W_{5}+\beta_{6}W_{6}+\beta_{7}A\times W_{1}$. $\beta_{A}$, the coefficient for the exposure was set to log(1.75). The interaction term $A\times W_{1}$ is included with coefficient $\beta_{7}$ set at 0 or 2 for the absence or presence of an extrapolation issue, respectively. When there is a lack of information on a covariate (e.g., $W_{1}$ with low prevalence) for certain levels of the exposure (leading to near‐positivity violation), the estimation of the exposure effect relies on extrapolating the observed effect. Biases will occur when the true exposure effect for the information that is lacking in the covariate differs compared to the fitted model that has been extrapolated into the values for the covariate that lacks information [39, 40, 41]. The presence of an interaction term between treatment A and covariate $W_{1}$ means that the effect of treatment is modified between groups of individuals, likely creating near‐positivity violation if there is imbalance in the distribution of $W_{1}$ between individuals. The rest of the coefficients were set to log(1.5). The distribution for the probability of the outcome is shown in Appendix Figure A1.

We simulated datasets of sample sizes $n_{obs}=\{200,1000\}$ representing small and large sample sizes, respectively, based on Leger et al. [38]. We chose a large enough sample of repetitions ($n_{reps}=1000$) such that we obtained a small enough Monte Carlo standard error without unfeasible computational time even for $n_{obs}=200$. The formula for the 95% confidence interval around the mean estimate is [42]:

$$p\pm 1.96*\sqrt{\frac{p(1-p)}{n_{\text{reps}}}}$$

Substituting p with the nominal coverage probability, 0.95 or 95%, the estimated coverage should fall between 93.6% and 96.4%.

### 3.3 Estimand, Methods, and Performance Measures

The estimand of interest was the ATE estimated by the difference in risks of the outcome between exposed and unexposed, $\hat{ATE}=\hat{\pi_{1}}-\hat{\pi_{0}}$, where $\hat{\pi_{a}}$ is the risk estimated in as the mean of $Q_{i}^{1}(a,W)$. The true values, $\pi_{1}$ and $\pi_{0}$, were estimated by averaging the values obtained from a univariate logistic model (the exposure as the only covariate), fitted from data sets generated above, except that the exposure A was simulated independently of the covariates W [38]. The true risk difference of the outcome between the exposed ($\pi_{1}$) and unexposed ($\pi_{0}$) was generated by averaging the true risk differences ($ATE_{i}$) across the repetitions.

We used four different estimation methods: TMLE and CVTMLE[Q], CVTMLE[Qg] and CVTMLE[all]. All estimation methods were used, by default, with the following algorithms within the SuperLearner: (i) stepwise selection, (ii) generalized linear modeling (glm), and (iii) a glm variant that included second‐order polynomials and two‐by‐two interactions of the main terms included in the models. We also included additional algorithms within the SuperLearner, such as Lasso (*glmnet* R package), Random Forest (*randomForest* R package), and Generalized Additive Models [all of which referred to as “RF”]. Therefore, the performances of eight methods were contrasted: TMLE, CVTMLE[Q], CVTMLE[Qg], CVTMLE[all], TMLE‐RF, CVTMLE[Q]‐RF, CVTMLE[Qg]‐RF, CVTMLE[all]‐RF. All simulated variables (i.e., $W_{1},W_{2},W_{3},W_{4},W_{5},W_{6},W_{7},W_{8}$) were included a priori for all estimation methods.

We assessed the performance of each method using measures of confidence interval coverage and relative bias [43]. The confidence interval coverage is the proportion of confidence intervals estimated around each repetition‐specific estimate $\hat{ATE}$ (i.e., ${\hat{ATE}}_{\text{low}}$, ${\hat{ATE}}_{\text{upp}}$) that include the true ATE. It is calculated as:

$$\begin{array}{ll} & \text{Coverage}=\mathrm{Pr}\left({\hat{ATE}}_{\text{low}}\leq \text{ATE}\leq {\hat{ATE}}_{\text{upp}}\right)\\ & \;\text{estimated by}\;\frac{1}{n_{\text{reps}}}\overset{n_{\text{reps}}}{\underset{r=1}{\sum }}1\left({\hat{ATE}}_{\text{low},r}\leq ATE\leq {\hat{ATE}}_{\text{upp},r}\right) \end{array}$$

Ideal confidence interval coverage is near 1−α, where α is usually chosen as 0.05. To reach nominal coverage, we expect that 95%‐confidence intervals would cover the true ATE in 95% of the repetitions.

The relative bias is the relative difference between the estimated ATE, $E[\hat{ATE}]$, and the true value of the ATE and is calculated as:

$$\text{Relative Bias}=\frac{E[\hat{ATE}]-ATE}{ATE}=\frac{\frac{1}{n_{\text{reps}}}\sum_{r=1}^{n_{\text{reps}}}({\hat{ATE}}_{r}-ATE_{r})}{\frac{1}{n_{reps}}\sum_{r=1}^{n_{reps}}ATE_{r}}$$

All analyses were performed in Stata statistical software (StataCorp, 2020, StataCorp LLC, College Station, TX). The Stata code to run the simulations is available at: https://github.com/mattyjsmith/CVTMLE We used the *eltmle* command to perform all methods, the development version is available at: https://github.com/migariane/eltmle [44]. Recent updates include the functionality to assess positivity violations via covariate balance tables. The command has been updated to perform cross‐validated TMLE[Qg], but it is a development version.

## 4 Results

**FIGURE 2:**
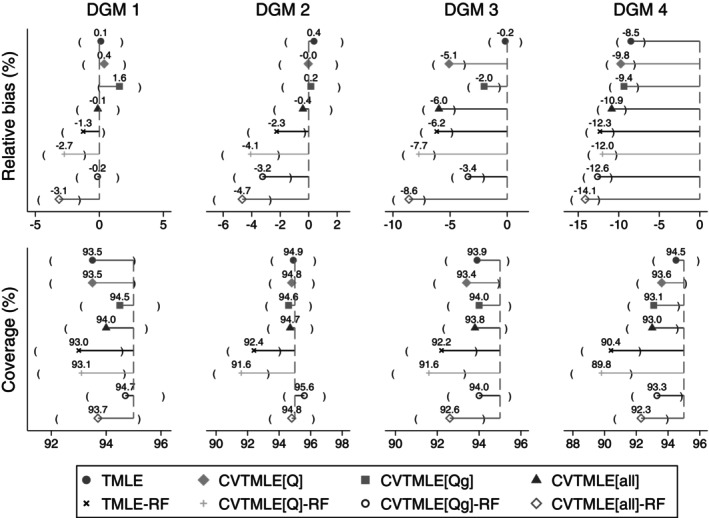
Relative bias and coverage of all TMLE and CVTMLE approaches under data‐generating mechanisms 1–4.

**FIGURE 3:**
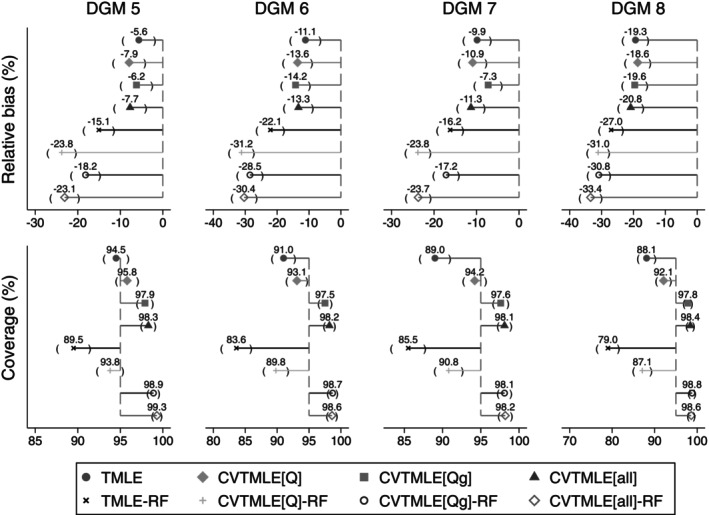
Relative bias and coverage of all TMLE and CVTMLE approaches under data‐generating mechanisms 5–8.

We report the performance measures for all simulated scenarios in Figures 2 and 3; the data‐generating mechanisms (DGM) are organized as described in Table 2.

**TABLE 2:** Summary of data‐generating mechanisms.

DGM	Sample size (N)	Prevalence of exposure	Extrapolation issue
1	1 000	50%	No
2	1 000	80%	No
3	1 000	50%	Yes
4	1 000	80%	Yes
5	200	50%	No
6	200	80%	No
7	200	50%	Yes
8	200	80%	Yes

### 4.1 Relative Bias

With large sample sizes (Figure 2), and no extrapolation issue (DGMs 1 and 2), there is negligible relative bias at less than 2% for TMLE, CVTMLE[Q], CVTMLE[Qg], and CVTMLE[all], and less than 5% for the same algorithms with Random Forests. With extrapolation issues (DGMs 3 and 4), there was some increase in relative bias. The relative bias was further increased when Random Forests were used in the Super Learner to between 1%–14%. Similar trends were observed for small sample sizes (Figure 3), except the relative bias was noticeably larger for DGM 6, 7, and 8, over 10%.

### 4.2 Coverage

With large sample sizes (Figure 2), and no extrapolation issue (DGM 1 and 2), there was approximately 95% coverage for all methods except those that include Random Forests as a package in the Super Learner. Generally, with an increasing severity of near‐positivity violation, TMLE or TMLE‐RF, and CVTMLE[Q]‐RF appear to perform worse. TMLE‐RF consistently showed undercoverage between 79% (DGM 8) and 92.4% (DGM 2). In small sample sizes (Figure 3), TMLE and TMLE‐RF consistently showed undercoverage, which was more noticeable with more extreme near‐positivity violations (DGM 6,7, and 8). CVTMLE[Q] showed good coverage, though slight undercoverage in DGM 8. CVTMLE[Qg], CVTMLE[Qg]‐RF, CVTMLE[all], CVTMLE[all]‐RF consistently showed an overcoverage (DGMs 5 to 8).

## 5 Empirical Example

We aim to study the effect of chemotherapy treatment initiation for patients diagnosed with diffuse large B‐cell lymphoma (DLBCL) between January 2014 and December 2017 on the probability of death at 6 months. We selected adult patients aged 18–85 years with a Charlson comorbidity score of 2 or less. Since treatment was not initiated on the day of diagnosis for all patients, we used a landmark time by which patients were assigned to be in the treated group or the untreated group. The treated group was defined as patients initiating treatment up to 21 days since diagnosis of DLBCL; those not treated within 21 days since diagnosis were considered the untreated group. The outcome was all‐cause death at 6 months from the landmark time, conditional on surviving 21 days since diagnosis; thus, the maximum follow‐up time was 6 months and 21 days since diagnosis. Models were adjusted for the following confounders: Age at diagnosis, sex, ethnicity (white/other), cancer stage (I/II/III/IV), Charlson comorbidity score (0, 1, or 2), and quintiles of the income domain of the deprivation score assigned to their small area of residence (1: least deprived, 2, 3, 4, 5: most deprived). Information on performance status, bulky disease, and presence of B symptoms was either not available or poorly recorded. There was no right censoring (loss‐to‐follow‐up) since we used national population‐based cancer registry data for all patients diagnosed with cancer in England, linked to death certification: Patients are assumed alive until their record matches a death record.

In the cohort, 3073 (22.2%) patients initiated chemotherapy treatment within 21 days of diagnosis, and 10,754 (77.8%) patients did not. At six months after the landmark time, 519 (16.9%) treated and 1442 (13.4%) untreated patients had died. We applied the eight approaches of TMLE described in this simulation study to estimate the ATE, measured as the risk difference in all‐cause 6‐month mortality between patients with DLBCL who initiated treatment within 21 days since diagnosis compared to those who did not initiate treatment.

We found that the risk of 6‐month all‐cause mortality is approximately 3% higher amongst those who initiated treatment within 21 days compared to those who did not, conditional on surviving 21 days since diagnosis of DLBCL (Table 3). Across the eight methods used in this analysis, the estimate of the ATE ranged from 2.84 (TMLE) to 3.17 (CVTMLE[Qg]‐RF). All eight methods agreed on statistical significance and had estimate agreement (i.e., estimates of the ATE from one method are contained within the 95% CI for each of the other methods).

**TABLE 3:** Risk of all‐cause mortality within 6 months (from landmark time of 21 days) between those who initiated treatment within 21 days since diagnosis of DLBCL and those who did not initiate treatment.

Method	ATE	SE	95% CI	*p*
Without random forests				
*TMLE*	2.84	0.0074	(1.39, 4.28)	0.0005
*CVTMLE[Q]*	2.90	0.0074	(1.45, 4.35)	0.0004
*CVTMLE[Qg]*	2.85	0.0074	(1.39, 4.31)	0.0005
*CVTMLE[all]*	3.00	0.0074	(1.55, 4.45)	0.0002
With random forests				
*TMLE*	3.11	0.0077	(1.59, 4.62)	0.0002
*CVTMLE[Q]*	3.05	0.0075	(1.59, 4.51)	0.0002
*CVTMLE[Qg]*	3.17	0.0079	(1.63, 4.71)	0.0002
*CVTMLE[all]*	3.01	0.0079	(1.46, 4.56)	0.0003

*Note:* Each of CVTMLE[Q], CVTMLE[Qg], and CVTMLE[all] used 10 folds during sample splitting.

Abbreviations: 95% CI, Confidence interval; ATE, Average treatment effect; SE, Standard error.

The distribution of patient characteristics was similar to DGM 2 or DGM 4 from the simulations, with a large sample size, approximately 80% prevalence of the exposure, and a possible interaction between initiation of treatment and stage at diagnosis on 6‐month mortality.

These results are in line with previous results showing that earlier treatment is associated with worse prognosis [45, 46, 47, 48, 49, 50]. Although counterintuitive that earlier treatment leads to a higher risk of mortality, this paradoxical effect can be explained through a lack of adjustment for the severity of disease. Highly aggressive DLBCL tends not only to have a higher risk of mortality but also to be treated earlier than less aggressive disease. Unless disease severity is adjusted for, paradoxical findings such as these are likely to occur.

## 6 Discussion

We found that combining targeted maximum likelihood estimation with cross‐validation (CVTMLE) improves coverage without adversely affecting bias in comparison to standard TMLE results, particularly in settings of small sample sizes and near‐positivity violations. In terms of bias and coverage, TMLE performs as well as CVTMLE in large sample sizes but suffers when the Donsker class condition is in question, with undercoverage in cases of small sample sizes with extrapolation issues, or unbalanced prevalence of the exposure.

It has been advocated that researchers should use a richly specified library of machine learning algorithms within the SuperLearner to maximise the performance of the estimation approach [51]. Previous research suggests that tree‐based methods, such as random forests, should be used with care because they tend to overfit the data [52]. In concordance, we found that the use of random forests led to a severe undercoverage when used with TMLE in all settings. If tree‐based methods must be utilized in the estimation step (i.e., due to the presence of heterogeneous treatment effects) [53], we advocate for the use of cross‐validation to optimize the estimation of the standard error and retrieve appropriate coverage, CVTMLE[Q], CVTMLE[Qg], and CVTMLE[Qg]‐RF led to coverages closest to 95% for most data‐generating mechanisms.

**FIGURE 4:**
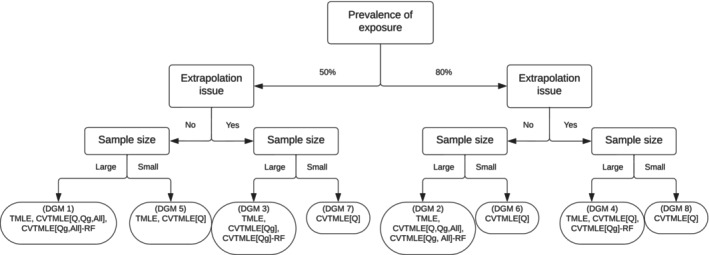
Decision tree for the appropriate choice of method given the scenarios (i.e., near‐positivity violation, sample size) that can cause the lack of differentiability of the influence curve and potentially violate the Donsker class condition.

As shown in this simulation study, the choice of the method to use is dependent on whether the data exhibits characteristics that could lead to a violation of the Donsker class condition. We provide a decision tree to guide the choice of estimation method in applied settings depending on the prevalence of the exposure, the finite sample size, and the presence of potential extrapolation and/or near‐positivity violations due to data sparsity (Figure 4). For example, in Branch (DGM 1) where there is 50% prevalence of the exposure, no extrapolation issue, and large sample size, our results suggest that either of TMLE, CVTMLE[Q, Qg, All] and CVTMLE[Qg, All]‐RF could be chosen to obtain a reasonably unbiased estimate of the ATE with optimal coverage. CVTMLE[Q] and CVTMLE[Qg] are suitable choices for most of the branches and can be the only appropriate choice, particularly in settings with near‐positivity violation and small sample sizes (such as in Branches DGM 6, 7, and 8). However, cross‐validation is computationally intensive, and if there are other methods (e.g., standard TMLE) that would perform the analysis to a similar degree of accuracy, then these other methods could be considered. Such instances occur with large sample sizes, where TMLE is least biased and within the optimal coverage range.

We generated data with simple outcome and exposure models to focus on and highlight the improvements in coverage when using cross‐validation with TMLE. Naimi et al. (2021) compared the performance of TMLE between simple and complex models [51]. In our simulation study, we did not include complex terms other than an interaction between the exposure and a variable causing the near‐positivity violation. Further studies are needed to explore the performance of these two methods in the context of data generated by complex models and heterogeneous treatment effects (i.e., inclusion of additional interactions, non‐linear, and time‐dependent effects). We speculate that methods employing additional algorithms (e.g., random forests) might perform better in terms of bias and, with cross‐validation, coverage. Moreover, we considered only binary variables for the outcome and exposure. The performance of these methods in settings with a continuous exposure or outcome requires further exploration: We speculate that the trends and patterns observed in this simulation study are generalizable to continuous outcomes and exposures, but this requires further research to confirm.

Doubly robust cross‐validated estimators have been developed to reduce overfitting and impose less restrictive complexity conditions on the machine learning algorithms used to estimate nuisance functions [17, 24, 54]. Sample splitting requires that the machine learning estimation of the nuisance parameters is fitted on a partition of the data set separate from the data used for calculating the target causal parameter. The role of the train and test samples can be swapped, which is called cross‐fitting. Single cross‐fitting only requires a division into training and prediction splits, but double cross‐fitting requires at least three splits of the data. Either cross‐fitting procedure can have different folds (e.g., 5 or 10) [17, 24, 27, 55]. Implementation of sample splitting procedures can be dependent on the chosen random number seed for random number generation that provides a particular split to the data. Solutions have been proposed elsewhere [56, 57, 58]; however, using a higher number of splits helps to avoid such dependency [59]. Previous research has shown that smaller sample sizes require an increase in the number of folds when performing the Super Learner [60]. This is to allow a sufficiently large training set to train the nuisance models. We did not alter the default setting of 10 folds used within the Super Learner, but the benefit of correctly specifying the number of required folds for cross‐validation within the Super Learner and the cross‐validation of TMLE is an area of ongoing research. We contrasted 5 and 10‐fold cross‐validation schemes and did not notice differences in performance between the various methods.

Compared to one‐step algorithms, TMLE is a more complex algorithm, making it less accessible to a lay audience. While TMLE is available in several software [61, 62], to our knowledge, the functionality to cross‐validate TMLE is limited to only R (*tmle* [63], *tmle3* [64]) and Stata (*eltmle* [44]). Importantly, TMLE R software defaults to CVTMLE[Qg]. Other packages exist that can be adapted to cross‐validate TMLE, such as Origami [65] for TMLE3 [64] in R, but tutorials are sparse. We used the *eltmle* command to perform all methods. The development version, including the CVTMLE[Qg] option, is available at: https://github.com/migariane/eltmle [44].

This study was limited to only one estimator, but other doubly robust estimators exist, such as augmented inverse probability of treatment weighting (AIPTW) and Double‐Debiased Machine Learning. We considered only TMLE‐based methods because (i) of their better stability, and (ii) we aimed to specifically investigate the undercoverage of TMLE [29]. CVTMLE helps to make the estimator consistent in larger samples; however, performance issues may still occur for finite samples [25]. For example, if the data violates the positivity assumption (i.e., the probability of being exposed, or unexposed, is too close to 0 or 1), which is more likely in smaller samples, then instability of the inverse weighting may occur in the targeting step. A simplistic approach is to truncate the propensity score at 0.975 and 0.025. However, collaborative‐TMLE (C‐TMLE) is another viable option [18, 66, 67]: C‐TMLE adaptively estimates the propensity score based on the outcome regression and mitigates practical positivity violations [52]. C‐TMLE has been recently developed that perform a model selection in estimating the propensity score model, which prevents the targeting step from introducing instability into the estimator of the outcome model. In this study, we focused on the comparison of TMLE and CVTMLE; further studies are needed to compare these other methods.

We observed that TMLE produces an underestimate of the coverage in settings with small sample sizes, the presence of an extrapolation issue, or imbalances prevalence of the exposure; however, combining cross‐validation with TMLE allows a consistent and reliable estimate of the coverage. The analysis of high‐dimensional data is an increasingly common activity for applied researchers, which often requires handling complex relationships between variables, and is likely to incur many of the data‐generating mechanisms employed in this simulation study. The implications of these findings suggest that it is not only important to check all necessary distributions (e.g., overlap plots) before estimating the effect of interest but that applied researchers should be cautious when choosing the appropriate method to analyze high‐dimensional data and strongly consider using cross‐validation, or similar, techniques to avoid issues with undercoverage that may occur in standard TMLE.

## 7 Conclusion

In conclusion, our simulation study reveals the benefits of incorporating targeted maximum likelihood estimation with cross‐validation in addressing coverage issues, particularly for small sample sizes and near‐positivity violations. Notably, the cross‐validation of the outcome model (CVTMLE[Q]) and of the outcome and treatment models, CVTMLE[Qg] yielded optimal coverage estimates. Our results underscore the importance of cross‐validation techniques, especially in the analysis of high‐dimensional data, cautioning researchers to consider cross‐validation to mitigate issues of undercoverage whenever TMLE or TMLE with RF is implemented.

## Author Contributions

The article arose from the motivation to understand how cross‐validated targeted maximum likelihood estimation performs in the presence of positivity violations. All authors developed the concept, and M.J.S. wrote the first draft of the article. M.J.S., R.V.P., M.A.L.‐F., and C.M. revised the manuscript. All authors read and approved the final version of the manuscript. M.J.S. is the guarantor of the article.

## Conflicts of Interest

The authors declare no conflicts of interest.

## Data Availability

The authors have nothing to report.

## Appendix A Tables and Figures

The probability of the outcome for 50% prevalence is shown in Figure A1A,C, and for 80% prevalence is shown in Figure A1B,D. A high extrapolation issue, created by an interaction between the exposure A and $W_{1}$ in the outcome model, is shown in Figure A1C,D, and leads to non‐parallel lines for the probabilities of the outcome by treatment group. There was no extrapolation issue generated in scenarios depicted in Figure A1A,B.

**TABLE A1:** Summary statistics of propensity scores by sample size, prevalence of the exposure “P(A=1)”, and exposure group.

			Propensity scores			n>0.975	
Sample size	P(A=1)	A	Min	Mean	Max	Mean	Range
200	50%	1	0.222	0.554	0.921	0.0	(0, 0)
		0	0.178	0.452	0.845	0.0	(0, 0)
	80%	1	0.513	0.812	0.981	2.2	(0, 9)
		0	0.510	0.752	0.931	0.0	(0, 2)
1 000	50%	1	0.182	0.554	0.945	0.0	(0, 1)
		0	0.143	0.453	0.902	0.0	(0, 0)
	80%	1	0.454	0.813	0.987	10.8	(2, 22)
		0	0.440	0.753	0.963	0.2	(0, 3)

*Note:* The mean of “n>0.975” is the average number of propensity scores, across the 1000 samples, that exceed the truncation value of 0.975. The range is the minimum and maximum number of propensity scores, across the 1000 samples, that exceed the truncation value of 0.975.
